# Improved Multi-Size, Multi-Target and 3D Position Detection Network for Flowering Chinese Cabbage Based on YOLOv8

**DOI:** 10.3390/plants13192808

**Published:** 2024-10-07

**Authors:** Yuanqing Shui, Kai Yuan, Mengcheng Wu, Zuoxi Zhao

**Affiliations:** 1 College of Engineering, South China Agricultural University, Guangzhou 510642, China; 20222168017@stu.scau.edu.cn (Y.S.); 20211168010@stu.scau.edu.cn (K.Y.); 13119046520wmc@stu.scau.edu.cn (M.W.); 2 Key Laboratory of Key Technology on Agricultural Machine and Equipment, South China Agricultural University, Ministry of Education, Guangzhou 510642, China

**Keywords:** flowering Chinese cabbage, small object, deep learning, ByteTrack, real-time detection

## Abstract

Accurately detecting the maturity and 3D position of flowering Chinese cabbage (*Brassica rapa* var. chinensis) in natural environments is vital for autonomous robot harvesting in unstructured farms. The challenge lies in dense planting, small flower buds, similar colors and occlusions. This study proposes a YOLOv8-Improved network integrated with the ByteTrack tracking algorithm to achieve multi-object detection and 3D positioning of flowering Chinese cabbage plants in fields. In this study, C2F-MLCA is created by adding a lightweight Mixed Local Channel Attention (MLCA) with spatial awareness capability to the C2F module of YOLOv8, which improves the extraction of spatial feature information in the backbone network. In addition, a P2 detection layer is added to the neck network, and BiFPN is used instead of PAN to enhance multi-scale feature fusion and small target detection. Wise-IoU in combination with Inner-IoU is adopted as a new loss function to optimize the network for different quality samples and different size bounding boxes. Lastly, ByteTrack is integrated for video tracking, and RGB-D camera depth data are used to estimate cabbage positions. The experimental results show that YOLOv8-Improve achieves a precision (*P*) of 86.5% and a recall (*R*) of 86.0% in detecting the maturity of flowering Chinese cabbage. Among them, mAP50 and mAP75 reach 91.8% and 61.6%, respectively, representing an improvement of 2.9% and 4.7% over the original network. Additionally, the number of parameters is reduced by 25.43%. In summary, the improved YOLOv8 algorithm demonstrates high robustness and real-time detection performance, thereby providing strong technical support for automated harvesting management.

## 1. Introduction

Flowering Chinese cabbage, a variety of *Brassica rapa* subspecies of the family Cruciferae, is one of the main cultivated vegetables in South China; the leaves of the plant can be used as food, the plant grows rapidly to a height of 20–30 cm within one month, and the stubble can be left to grow repeatedly, so it has good economic characteristics [1]. Flowering Chinese cabbage is preferred by many people as it is a vegetable rich in vitamins and other nutrients [2]. Currently, the main stem and leaf crops are mainly hand-picked after manual inspection [3]. It is worth noting that the maturity time of flowering Chinese cabbage often varies even in the same carefully cultivated batch, which requires frequent and accurate assessment of the maturity of each plant to implement timely hand picking. However, the traditional hand-picking method is not only time-consuming, labor-intensive and inefficient, but also costly, which severely limits the development of Chinese flowering cabbage industry. Mechanical harvesting and picking can significantly reduce labor intensity and production costs. The combination of image processing and machine vision technology can realize accurate real-time detection of Chinese flowering cabbage to understand its maturity, which can provide the necessary support for automatic harvesting machines and is one of the cores of future mechanical harvesting technology [4].

The rapid detection of flowers and fruits is crucial for the development of automated flower and fruit harvesting robots [5,6], and the prerequisite for accurate and selective harvesting is the efficient detection of targets using computer vision, which is a challenging problem [7]. Traditional machine vision techniques focus on intuitive features as the basis for flower and fruit recognition. With the continuous development of traditional target detection algorithms, the performance improvement of existing traditional algorithms has become extremely limited [8]. However, the current research of maturity detection in leafy vegetables, particularly Chinese cabbage, faces unique challenges. Compared to the obvious ripening characteristics of fruits, vegetables have very little variation among individuals at different growth stages. Leafy vegetables, on the other hand, have fewer detectable traits and pose significant hurdles for non-destructive maturity evaluation [9]. Consequently, there is a notable dearth of research focusing on non-invasive pre-harvest maturity assessment of Chinese cabbage. Drawing inspiration from China’s established industrial norms for grading flowering Chinese cabbage, the discernible changes in the morphology and coloration of cabbage buds across maturity stages emerge as crucial indicators for maturity assessment [10]. Thus, there is a pressing need to develop innovative approaches that can overcome the limitations of traditional methods and harness these indicators for accurate and timely maturity detection in leafy vegetables.

Pornpanomchai et al. [11] utilized the features of herbaceous flowers to design classification algorithms for herbaceous flower detection and recognition. Lin et al. [12] proposed a support vector machine (SVM) that relies on silhouette information SVM to overcome the problem of light variations and low color contrast for the recognition of citrus and tomato fruits. Guru et al. [13] segmented flower images by removing the background through a threshold-based method, specifically utilizing color texture moments, gray-level co-occurrence matrix and Gabor responses for flower recognition and classification purposes. Guo et al. [14] used a customized hyperspectral dataset that integrates both spectral and textural attributes of strawberries to assess their ripeness, achieving a classification accuracy of more than 85%. Although some problems can be solved based on traditional methods, traditional target detection algorithms still have certain limitations: first, the generation of candidate regions generates a large amount of redundancy, which significantly increases the computational burden and results in a waste of resources; second, in the face of variable and complex backgrounds, it is difficult to accurately capture the key semantic information by relying on low-level visual feature descriptors, which greatly restricts the applicability and generalization ability of the algorithms in complex environments [15]. Therefore, when facing complex and changing natural scenes, such as the high complexity of the background, severe mutual occlusion among targets and uneven distribution of lighting conditions, traditional target detection algorithms are often difficult to fully satisfy the requirements of high accuracy and robustness.

In the current agricultural field, deep learning technology has become a mainstream method in the field of flower, fruit and vegetable detection in view of the strong robustness and generalization ability it exhibits [16,17]. Compared to traditional machine vision techniques, deep learning techniques learn more abstract and representative features and semantic information by constructing deep neural network models, which improves the generalization ability of the models as well as the accuracy and speed of object detection in complex environments [18]. Tian et al. [19] proposed an instance segmentation model using U-Net backbone to improve Mask Scoring ReCNN for detecting and segmenting apple blossoms with three different growth state levels. Lin et al. [20] improved an algorithm for strawberry blossom detection based on Faster R-CNN coupled with migration learning techniques. Strawberry flower detection in outdoor environments was realized in the face of flower overlapping and complex backgrounds. Chen et al. [21] proposed a strawberry flower and fruit detection system based on Faster R-CNN with high-resolution ortho images reconstructed from UAV images. Qi et al. [22] used a highly converged, lightweight deep learning architecture for tea chrysanthemum flower detection based on YOLO (TCYOLO) to detect tea chrysanthemum flowers in complex natural environments. Zeng et al. [23] presented the THYOLO algorithm, a lightweight solution tailored for fast tomato fruit detection and ripeness categorization. By incorporating MobileNetV3 and optimizing the neck channels, they achieved a compact model size of 6.04 MB, but only with a recognition speed rate of about 26.5 FPS. Summarizing the previous research of these methods on flower, fruit and vegetable detection, there are still some challenges: first, most of the models lack the required lightweight and efficient features. Second, the focus is on the detection speed, and the target size variation is not significant. Most of them concentrate on scenarios involving a small number of targets in close-range imaging, where the restricted count and substantial size of the targets significantly diminish the complexity of detection.

In the actual agricultural production process, high-precision vision sensors, integrated atop picking robots, are capable of omnidirectionally capturing image information of multiple target objects within the operational area, owing to their expansive field of view. However, the significant variation in target sizes arising from varying imaging distances poses challenges in accurately detecting small targets. Furthermore, given the high-density planting pattern inherent to flowering Chinese cabbage crops, the overlapping and occlusion of flowers and leaves complicate the task of distinguishing detection targets from the background. Meanwhile, the dynamic movement of the camera at different operating distances not only leads to instantaneous changes in the shape and size of the target objects in the field of view, but also raises the accuracy and robustness criteria that the detection algorithms need to fulfill in response to such dynamic environmental changes. In practical operation scenarios, it is particularly important to realize efficient and accurate detection of multi-size and multi-targets in view of the possible efficiency difference between robot picking speed and real-time detection processing speed.

Accordingly, this study is devoted to overcoming the detection challenges inherent to multi-size and multi-target scenarios and to achieve non-destructive maturity detection. To this end, it proposes an innovative flower detection and localization network for flowering Chinese cabbage. The network integrates the color, morphology and other key features of the flowers to accurately determine the maturity status of flowering Chinese cabbage. To achieve real-time detection in video, we integrated the ByteTrack tracking algorithm and introduced the RealSense D435i depth camera (Intel D435i depth camera is from Intel Corporation, Santa Clara, CA, USA) to extract comprehensive depth data, subsequently acquiring the precise 3D position of the detection target within the camera coordinate system. To ensure the broad representativeness and accuracy of the research data, a flowering Chinese cabbage dataset was meticulously constructed, containing multiple sizes and target categories, and targets were labeled with great care, including flowers and buds. Regarding the model design, a comprehensive examination was conducted of the principles and characteristics of the analytical attention mechanism, multi-feature fusion mechanism and loss function. Thereafter, a targeted optimization of the YOLOv8 network architecture was undertaken. A series of experiments demonstrated that the enhanced network exhibited markedly enhanced performance across multiple dimensions. Specifically, the network demonstrated the capacity to efficiently and accurately recognize a range of flowering Chinese cabbage targets, while also exhibiting remarkable stability and robustness in addressing multi-size and high-density target detection tasks. Notably, the network exhibited enhanced recognition accuracy and a reduced false detection rate in the context of small target detection, which is a crucial advancement for enhancing the precision and efficiency of automated agricultural harvesting.

## 2. Experimental Result and Analysis

### 2.1. Experimental Platform and Parameter Settings

All experiments conducted in this study were performed on the same platform of computer. The hardware configuration consisted of a Windows 11 Professional operating system, 13th generation processor Intel^®^ Core i5-13400F@2.50 GHz, 32 G DDR5 RAM and NVIDIA GeForce RTX 4070 with 12 GB (NVIDIA GeForce RTX 4070 GPUs is from NVIDIA Corporation, Santa Clara, CA, USA) of memory. The programming language utilized was Python 3.10, and the development framework employed was PyTorch 2.1.1. Furthermore, CUDA 12.1 was employed to accelerate the training process. The input image size was configured to 640 × 640, with a batch size of 16. The SGD optimizer was employed, and both the initial and termination learning rates were set to 0.01. After 100 epochs of training, the optimal weight file was obtained and used for model evaluation.

### 2.2. Comparison Experiments of Different Models

This experiment assesses the performance of several advanced target detection models on datasets collected in natural environments. The models include Faster R-CNN, SSD, RetinaNet, YOLOv5, YOLOv8, YOLOv9 and RE-DETR [24,25,26,27,28,29,30]. A comparison of the performance of these models in detecting the maturity of flowering Chinese cabbage is presented in Table 1, which provides specific details of the comparison.

Faster R-CNN demonstrates remarkable recall in identifying the ripeness of flowering Chinese cabbage, suggesting that it can efficiently minimize the number of missed targets. However, its detection precision is relatively low, which may be due to the model’s two-stage detection method, which, while capable of mitigating missed detections, is deficient in establishing a feature pyramid and integrating shallow feature information, resulting in a lack of precision in the details of the target, with mAP_50_ of 80.1% and a precision rate of only 70.7%.

In contrast, SSD enhances the precision of detection by integrating the predictive outcomes of diverse feature maps with varying resolutions, thereby enabling the model to more accurately align targets with the diverse shapes and sizes of flowering Chinese cabbage. However, in comparison to the two-stage detection approach, the recall rate of SSD exhibits a notable decline, leading to a comparatively low mAP_50_ of 75.7% among all evaluated models.

RetinaNet effectively extracts the feature information of flowering Chinese cabbage through a multi-stage feature fusion method, which, in turn, improves the detection accuracy. Considering the issue of positive and negative sample imbalance, which is prevalent in one-stage target detection methods, RetinaNet employs the Focal Loss function to achieve a more balanced outcome, thereby markedly enhancing its performance. In the comparative modeling experiments, RetinaNet exhibits a notable improvement of 87.3% in mAP_50_ compared to the previous two.

YOLOv5 is based on the feature pyramid, which makes full use of the feature information of different layers by introducing the CSPnet structure. To extract features, the feature layer backbone is divided into two distinct branches. which are extracted separately and then fused, thus making full use of the feature information of different layers. The deepening of the network layer further improves the learning ability of the model, and the utilization of residual blocks mitigates the issue of gradient vanishing that arises from deep networks. Nevertheless, YOLOv5 attains 83.3% and 83.1%, which correspond to recall and precision, respectively, on the task of detecting the maturity of flowering Chinese cabbage, indicating that there is still room for further improvement.

However, YOLOv8 uses a new C2F module to achieve further lightweighting, changing the head part from a coupled head to a decoupled head and adopting an anchor-free strategy to solve the conflict between classification and regression tasks. This optimization allows its mAP_50_ to reach a good value of 88.9%.

YOLOv9 incorporates the Generalized ELAN (GELAN) architecture to achieve optimized parameters, computational complexity, accuracy and inference speed effects. The concept of Programmable Gradient Information (PGI) is introduced, which helps in generating reliable gradients through assisted reversible branching and ensures that the deep features retain the key features needed to perform the target task. However, these changes failed to allow the model to show higher recall and precision in the difficult target detection task on this dataset, with a mAP_50_ of 81.1%.

The RT-DETR model achieves real-time target detection by optimizing the encoder and introducing a new attention mechanism. It is able to provide a high frame rate while maintaining a high precision. However, this model did not show better precision, recall and mAP_50_ in the dataset of this study, with results of 66.7%, 66.9% and 68.9%, respectively. As a Transformer model well balances the detection speed and accuracy, it will be advantageous in real-time detection, but it also occupies more computational resources.

The YOLOv8-Improved model exhibits optimized performance in feature extraction, fusion methodology and loss function, resulting in the best overall performance. Specifically, it achieves a recall rate of 86.5% and a precision of 86.0%, ultimately yielding the highest average precision in all comparative experiments with a mAP_50_ of 91.8%. Additionally, its comprehensive evaluation index, F1, is 0.86.

**Figure 1:**
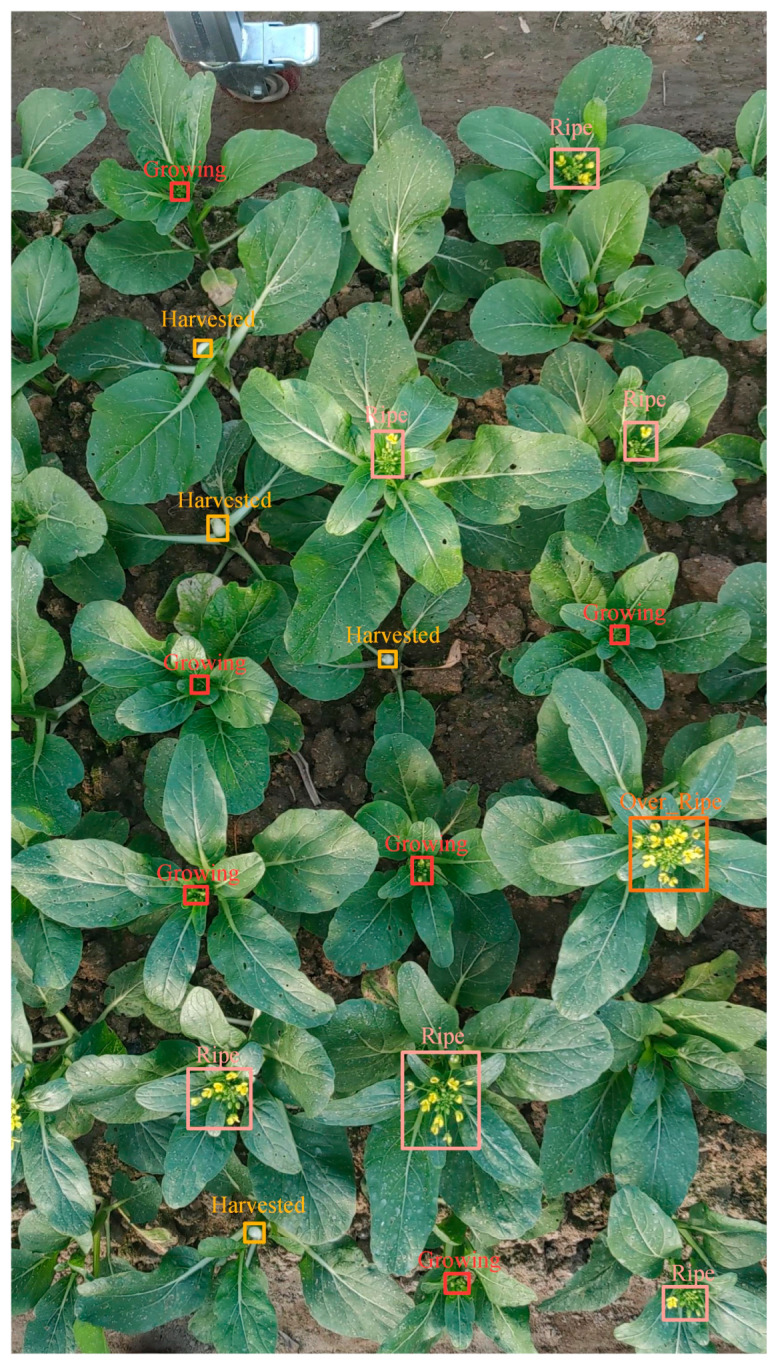
Original test image of detection sample.

Figure 1 represents the original sample image with annotation, with a smaller and partially obscured portion of the flowering Chinese cabbage. The original label contains 17 targets of Chinese cabbage.

**Figure 2:**
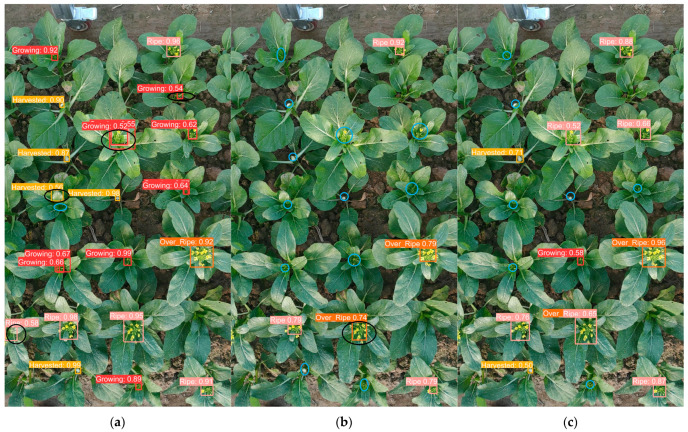

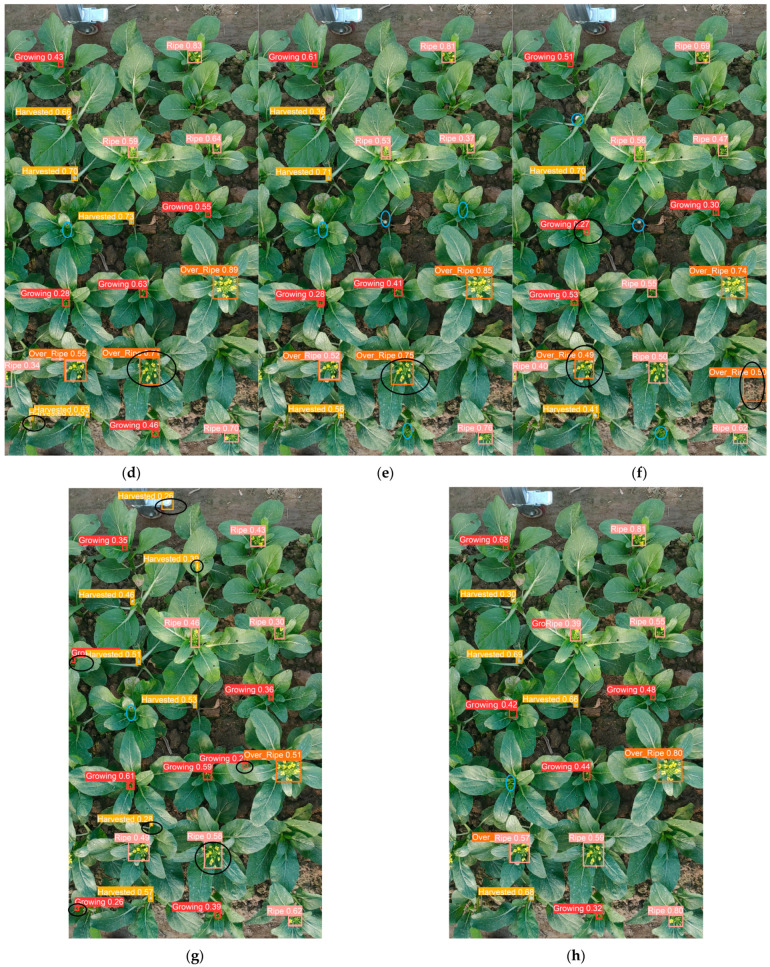
Prediction results under sunlight of different models: (**a**) Faster R-CNN; (**b**) SSD; (**c**) RetinaNet; (**d**) YOLOv5; (**e**) YOLOv8; (**f**) YOLOv9; (**g**) RT-DETR; (**h**) YOLOv8-Improved (Ours).

Figure 2 illustrates the performance of various models in detecting outcomes under a sunlit environment. The blue circle denotes the area where a detection was missed, while the black circle represents the region where a false detection occurred. Of the models evaluated, YOLOv8-Improved demonstrated the most optimal detection performance, as illustrated in Figure 2h, exhibiting a minimal number of missed detections and false positives. As shown in Figure 2d, YOLOv5 also demonstrated satisfactory detection performance, albeit with a slight increase in the number of false detected targets in comparison to YOLOv8-Improved. As seen in Figure 2b–f, respectively, one-stage detection models, SSD, RetinaNet, YOLOv5, YOLOv8 and YOLOv9, exhibited a tendency to miss certain regions, with SSD demonstrating the greatest propensity for this phenomenon. Additionally, the detected targets often correspond to anchor frame sizes that are incongruent with the actual dimensions of the objects in question. In comparison, Faster R-CNN as a two-stage detection model, as shown in Figure 2a, exhibits a lower miss rate than the one-stage models, yet it also displays a slight proclivity for false detection targets.

**Figure 3:**
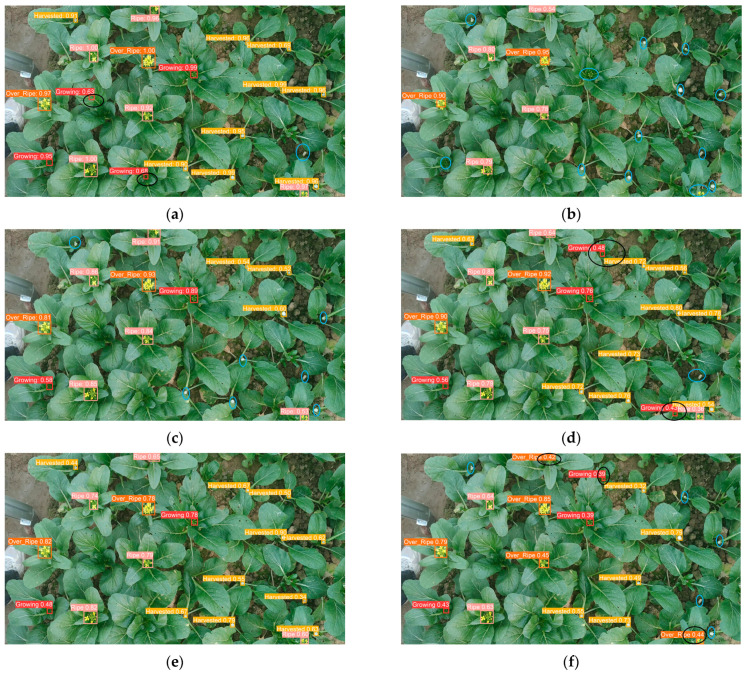

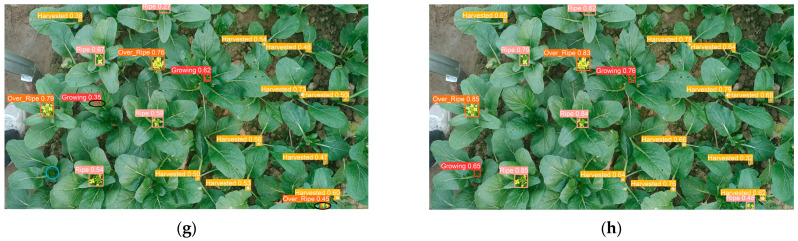
Prediction outcomes under cloudy conditions of different models: (**a**) Faster R-CNN; (**b**) SSD; (**c**) RetinaNet; (**d**) YOLOv5; (**e**) YOLOv8; (**f**) YOLOv9; (**g**) RT-DETR; (**h**) YOLOv8-Improved (Ours).

Figure 3 illustrates the performance of various target detection models in detection scenarios under cloudy conditions. While RT-DETR, in Figure 3g, demonstrates efficacy in processing certain targets, it is observed that some targets are incorrectly identified. In the one-stage detection model, as shown in Figure 3b,c,f, the SSD, RetinaNet and YOLOv9 models exhibit a greater tendency to miss regions during detection, with the SSD model demonstrating particularly notable deficiencies in this regard. In contrast, in the two-stage detection model, as seen in Figure 3a, Faster R-CNN demonstrates a lower leakage rate and exhibits superior detection performance compared to many one-stage models. However, a minor proportion of false detection targets are also observed. All models demonstrate robust performance in the detection of larger targets. Nevertheless, when confronted with smaller targets, the detection capabilities of the different models exhibit notable disparities.

### 2.3. Ablation Experiments

**Figure A1:**
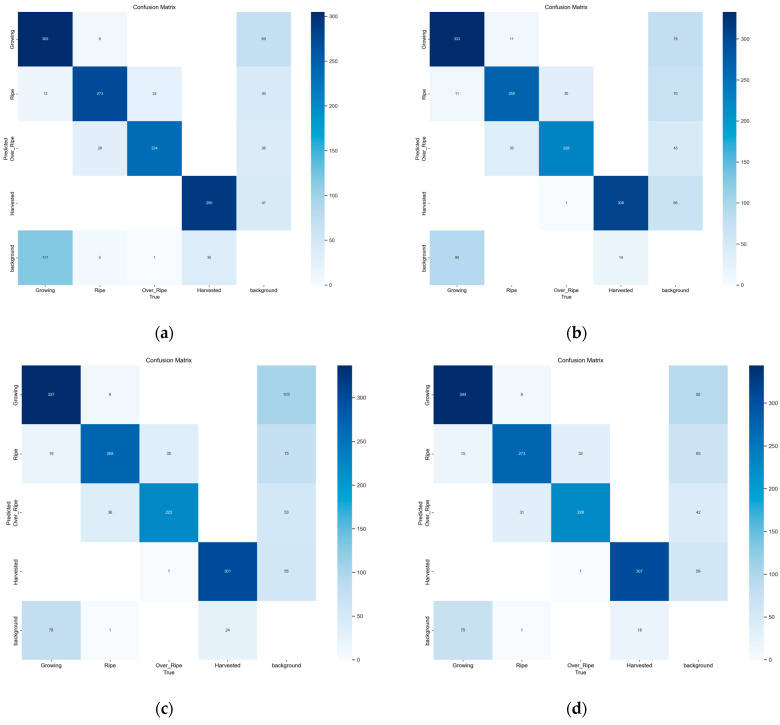

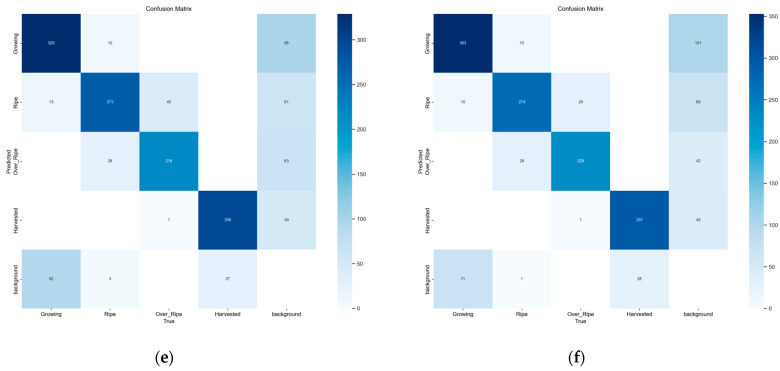
Confusion matrices: (**a**) YOLOv8; (**b**) YOLOv8-W; (**c**) YOLOv8-WX; (**d**) YOLOv8-WY; (**e**) YOLOv8-WZ; (**f**) YOLOv8-Improved.

Four enhancements are made to the original YOLOv8n model (W: adding a small object detection layer; X: adding MLCA mechanism to the C2F structure and applying it to the backbone; Y: using BiFPN as a neck network; and Z: replacing the original loss function with Wise-inner-IoU. In this context, X, Y and Z all add a small object detection layer). Each improved module except module W is separately incorporated into the basic model for ablation experiments, aimed at demonstrating the performance of the enhanced modules. The results are presented in Table 2. The confusion matrices of each model are detailed in Figure A1 of Appendix A.

**Figure 4:**
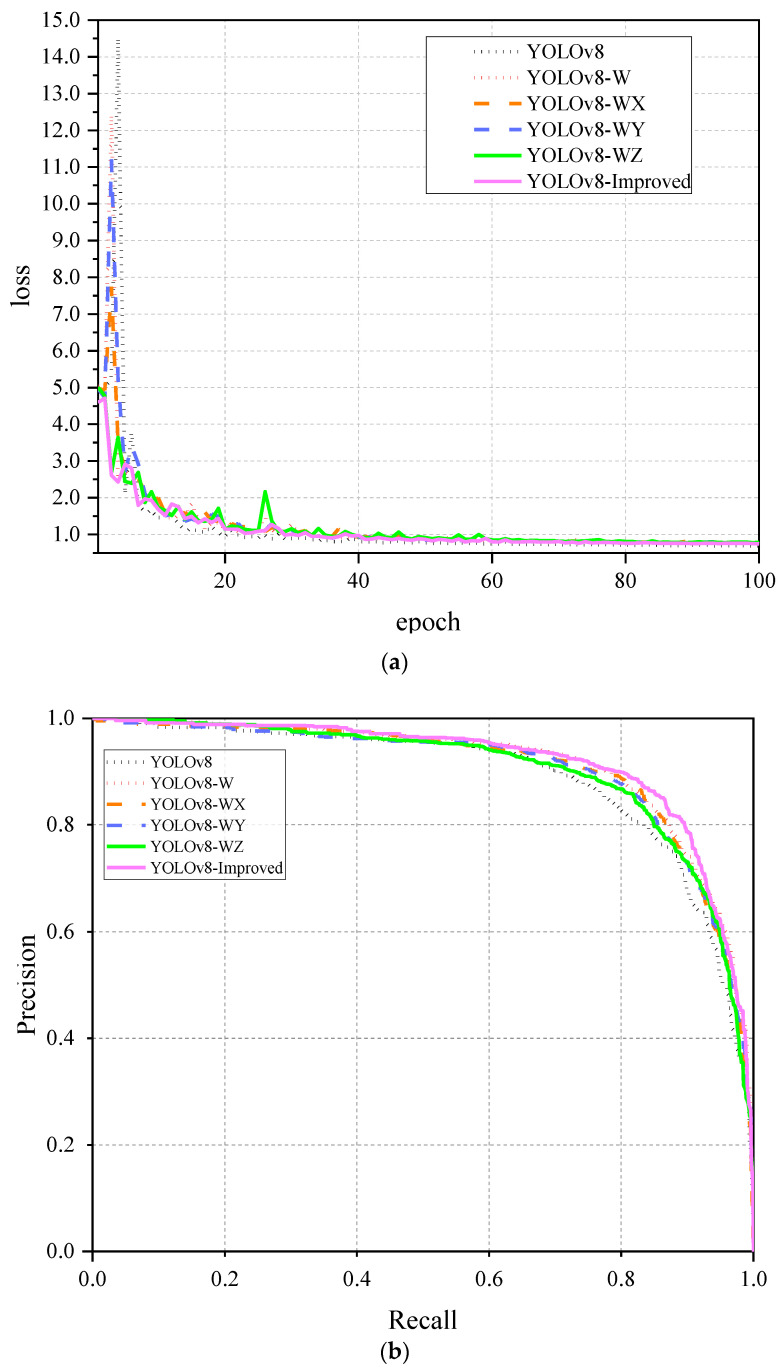
Ablation experiments: (**a**) Loss curve; (**b**) Precision-recall curve.

**Figure 5:**
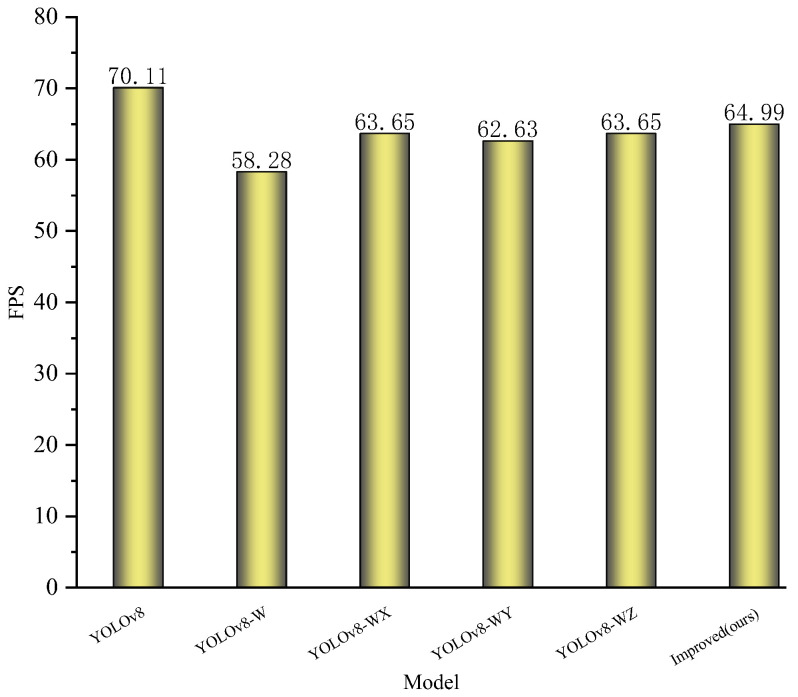
FPS for each model.

When a small object detection layer was added to the model, the mAP_50_ improved by 1.2%. After incorporating the MLCA mechanism with the C2F module into the base model, the mAP_50_ increased by 1.8%. The inclusion of the attention mechanism effectively enhanced the feature extraction ability of the network and suppressed the interference of useless information. The mAP_50_ increased by 2.5% after replacing PAN as a neck network with BiFPN. Replacing CIoU with Wise-Inner-IoU increased the mAP_50_ by 1.4% at this point. Finally, the best performance was exhibited after combining the small object detection layer, C2F-MLCA, BiFPN and Wise-Inner-IoU. As a result, Params was reduced by 25.34%, while precision, recall, mAP_50_ and mAP_75_ improved by 1.6%, 2.7%, 2.9% and 4.7%, respectively. Figure 4a shows the loss curve on the training set, while Figure 4b shows the P-R curve on the validation set. This observation suggests that the combination of MLCA, BiFPN and Wise-inner-IoU effectively reduces the loss values and enhances the detection performance. The FPS data of each model in the ablation experiments are shown in Figure 5; due to the increase in the computation and the slowing down of the inference speed, the YOLOv8-Improved model experiences a minor reduction in FPS when compared to the baseline model. This decrement, however, remains within an acceptable threshold for a real-time detection model capable of processing over 60 frames per second.

#### Attention Mechanism

In this study, for the YOLOv8-Improved model, the MLCA mechanism is introduced, with focus on the effects of its parameter “*ks*” (i.e., Size) which represents the number of blocks in the W or H dimension, and the weights (i.e., Weight) when global and local branches are summed up, as detailed in Section 3.2.2, on the model performance. By adjusting different combinations of the two parameters, the role of the attention mechanism on the model’s precision, recall and mAP_50_ metrics is systematically analyzed. Table 3 represents the experimental results.

The experimental results show that when the size is set to 5 and the weight is set to 0.5, the model exhibits optimal comprehensive performance, with precision reaching 86.5% and mAP_50_ as high as 91.8%, indicating that this configuration performs excellently in accurately recognizing and localizing targets. Although recall reaches a peak of 87.4% for size = 3, weight = 0.5 and size = 5, weight = 7 conditions, in the overall evaluation, the significant improvement of the combination of (5, 0.5) on mAP_50_ makes it the optimal configuration.

The size of *ks* is directly correlated with the quantity of spatial information introduced as a basis for model reference. In consideration of the characteristics of the sample data in this experiment, the 5 × 5 *ks* size is identified as a superior option, striking a balance between the sufficiency and necessity of spatial information. An excessively large *ks* may result in the introduction of superfluous spatial information, which impedes the model’s capacity to discern pivotal target characteristics. Conversely, an insufficiently small *ks* may lead to an inadequate extraction of spatial information, which, in turn, affects the accuracy of detection.

The local weight parameter serves to regulate the weight allocation when local features are fused with global features. The experimental results demonstrate that the model performance is optimal when this parameter is set to 0.5, indicating that a balanced combination of the two types of feature information can enhance the detection effect more effectively on this dataset. This verifies the superiority of the average reference strategy.

### 2.4. Application Performance of Tracking

In this study, we integrated the YOLOv8-Improved model with the ByteTrack tracking algorithm to develop an efficient and robust system for the detection of the maturity of flowering Chinese cabbage in dynamic scenes. The model is capable of continuous and stable tracking of the detected targets in complex dynamic environments, effectively mitigating the issue of target loss due to occlusion, rapid movement, or illumination changes.

In the design, particular emphasis was placed on the collection and processing of data. By setting the resolution of the RGB-D camera to 1280 × 720 and maintaining a frame rate of 30 fps, we ensured the high quality and smoothness of the video stream, which provided a solid foundation for the subsequent model detection. Furthermore, synchronizing the color stream of the depth camera with the depth stream was a critical step. This ensures that precise corresponding depth information is obtained for each frame, which is essential for subsequent accurate positioning and analysis of flowering Chinese cabbage maturity in three-dimensional space [31].

**Figure 6:**
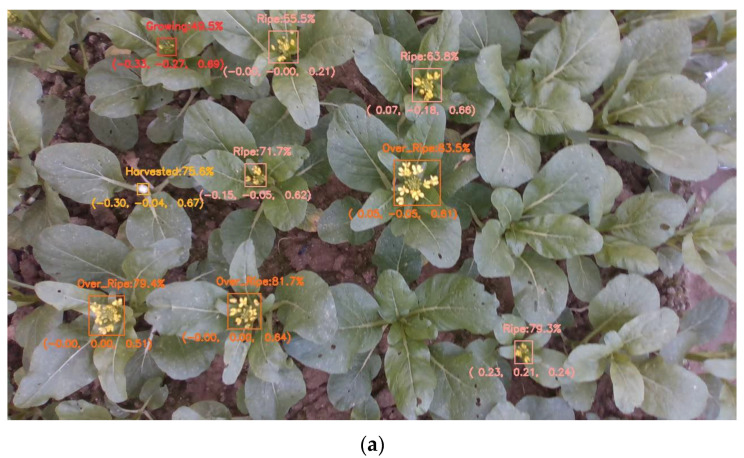

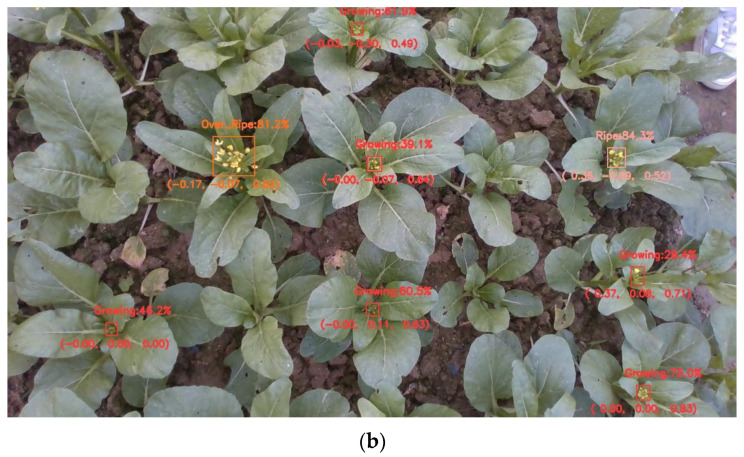
Results of ByteTrack. (**a**) Tracking result frame 1, (**b**) Tracking result frame 2.

As illustrated in Figure 6, the system demonstrated the capacity to accurately identify flowering Chinese cabbage at varying degrees of maturity, concurrently providing precise coordinates of every detected target in the coordinate system of the camera. The implementation of this functionality facilitates a more comprehensive analysis of the growth status of flowering Chinese cabbage in three-dimensional space, thereby providing robust technical support for precision agriculture management. By comparing the spatial distribution and growth trend of flowering Chinese cabbage at different maturity levels, agricultural management can develop a more scientific picking plan and enhance the efficiency of agricultural production.

## 3. Materials and Methods

### 3.1. Data Acquisition

**Figure 7:**
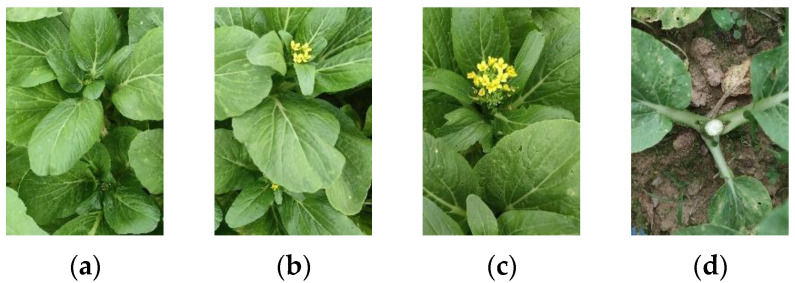
Different growth states of sample: (**a**) Growing, (**b**) Ripe, (**c**) Over-Ripe, (**d**) Harvested.

The dataset utilized in this study was procured from a field of flowering Chinese cabbage in its natural environment. The images were captured between October and November 2023 at South China Agricultural University in Guangzhou City, Guangdong Province, China, utilizing a specifically designed data acquisition platform with adjustable height. A mobile phone camera, specifically the OPPO Find X2 (OPPO Find X2 is from OPPO Guangdong Mobile Communications Co., Ltd., Dongguan, Guangdong, China) with a resolution of 3840 × 2160, was used for image capture, complemented by an Intel D435i depth camera for select research components. The camera, positioned at a height of 0.8 to 1 m from the plant canopy, was mounted on the platform and moved at a constant speed of approximately 0.15 m/s. The dataset comprised 1016 images, encompassing 11,970 target samples of flowering Chinese cabbage, capturing various stages from germination to flowering and maturity. The data collection spanned different time periods, including mornings and afternoons, under varying light conditions ranging from soft early morning light to intense midday sun and slanting evening light, to ensure the diversity of experimental data. The dataset was labeled and randomly divided into training, validation and test sets in a 7:2:1 ratio. The specific number of data samples is presented in Table 4, and an illustration of the data types is provided in Figure 7. To guarantee the training generalization and efficiency of the model, the image resolution of the training set was uniformly adjusted to 640 × 640.

### 3.2. YOLO v8-Improved Model

#### 3.2.1. YOLO·v8 Network Structure

As a one-stage detection algorithm, the YOLO series of networks is distinguished. Among the YOLO networks, YOLOv8 [24] is regarded as a particularly effective network. YOLOv8 consists of backbone, neck and head. The backbone network adopts a similar structure to YOLOv5, which improves the original CSPLayer module into a C2F module, which consists of a cross-stage partial bottleneck structure with two convolutional layers, which effectively facilitates the fusion of high-level features with contextual information [25]. A spatial pyramid structure (SPFF) featuring multiple size pooling windows is used at the conclusion of the backbone, aiming to further enhance its capacity for feature extraction. YOLOv8 employs a dual feature fusion strategy of FPN (Feature Pyramid Network) + PAN (Path Aggregation Network) as the neck network to achieve multi-scale feature extraction and enrich the detection of small targets. The head network, on the other hand, utilizes an anchor-frame-free model with a decoupled head structure to handle the regression and classification tasks separately, while the CIoU (Complete-IoU) [32] and DFL (Distribute Feature Loss) [33] loss functions are employed for closed frame prediction. Compared to the anchor-frame-based method, the number of parameters is reduced and better adapted to small targets with large shape differences.

**Figure 8:**
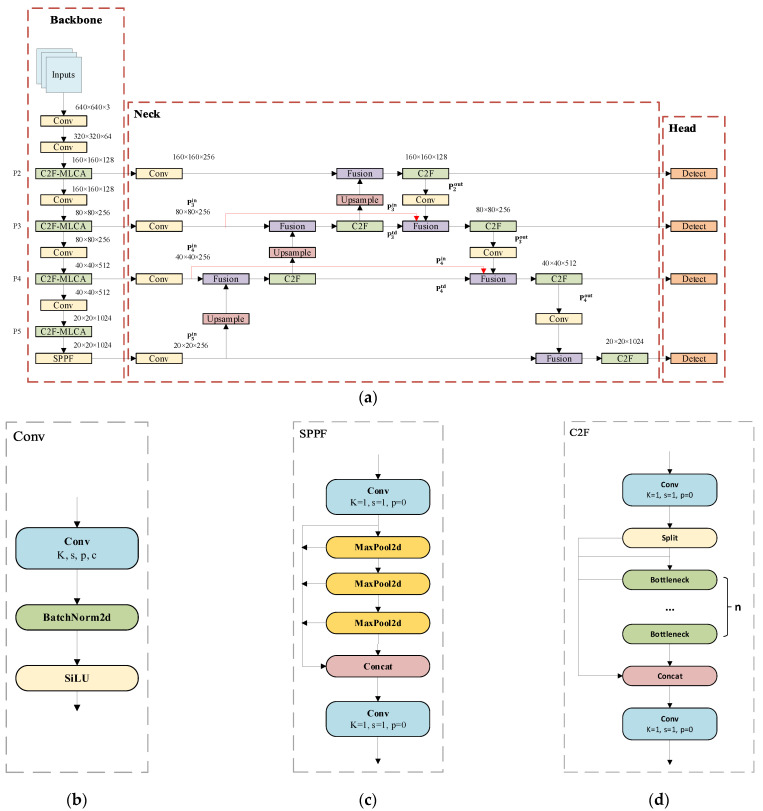

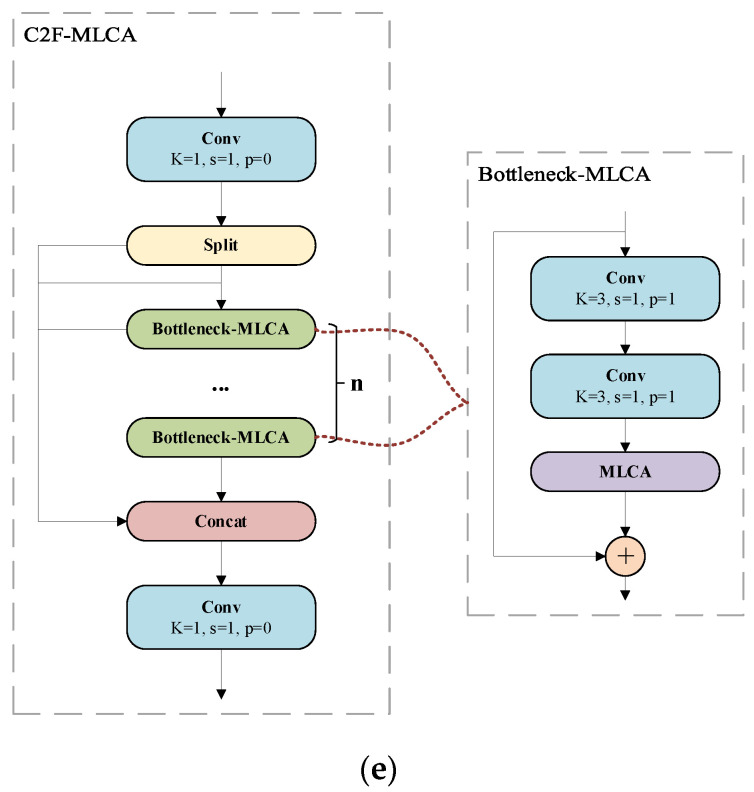
YOLOv8-Improved network architecture and modules: (**a**) YOLOv8-Improved; (**b**) Conv; (**c**) SPPF; (**d**) C2F-MLCA; (**e**) C2F.

To address the challenges of small size, high target similarity, overlap and occlusion faced in flowering Chinese cabbage flower detection, this study makes targeted improvements based on YOLOv8. Specifically, we introduced the C2F-MLCA module in the backbone network to enhance the feature expression capability; optimized the neck network structure with BiFPN (Bidirectional Feature Pyramid Network) for more effective fusion of multi-scale features; replaced the loss function with Wise-Inner-IoU to improve the accuracy of bounding box prediction; and added a detection layer output for small targets to the head network to better accommodate the detection needs of flowering Chinese cabbage. Figure 8 demonstrates the improved YOLO network framework. Next, the specific implementation details of these improvements are elaborated.

#### 3.2.2. BiFPN

**Figure 9:**
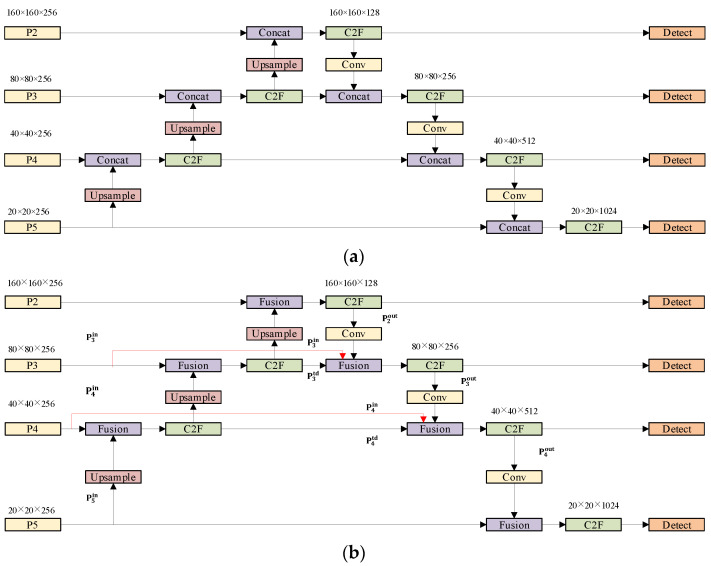
Neck network architecture: (**a**) PAN; (**b**) BiFPN.

To optimize the target detection architecture, we augment the existing P3, P4 and P5 detection layers with a P2 layer. The P2 layer has a narrow receptive field and is specialized for recognizing small target objects. When integrated with the BiFPN neck, it enables seamless bidirectional feature data exchange between layers. This multi-scale feature fusion technique enhances the network’s ability to localize small targets because the relevant features are prominently located in lower-level, high-resolution maps. The addition of P2 enhances the BiFPN’s ability to merge multiple levels of features, allowing the network to better integrate details from lower levels and abstract semantics from higher levels. In particular, the bidirectional information flow within the BiFPN ensures comprehensive contextual evaluation during feature integration, which is important for distinguishing visually similar targets. BiFPN employs a weighted feature fusion mechanism to integrate features of disparate levels. During the training process, the fused weights are learned, thereby ensuring optimal feature integration [34]. The structure of this feature fusion network is shown in Figure 9b. The majority of neck networks employ a straightforward aggregation approach for the integration of disparate input features, lacking any form of differentiation. However, this results in an uneven contribution to the fused output features, due to the inherent differences in resolution between the input features. In BiFPN, the input to the neck is given a multi-scale feature list ${\vec{P}}^{in}=(p_{l1}^{in},p_{l2}^{in},\ldots )$, where $p_{l1}^{in}$ denotes the features of the $l_{i}$ layer. The BiFPN transformation aggregates disparate features to produce a new feature list ${\vec{P}}^{out}=f({\vec{P}}^{in})$ as output.

Given the varying resolutions of the input feature maps, there is a natural variability in their contributions to the output feature maps. To optimize this fusion process, BiFPN introduces a more fine-grained strategy, in contrast to the previous traditional approach that simply treats all input features as equal weights. It assigns unique weight parameters to each input feature, which are adaptively learned during the training phase of the network, and the model automatically assigns appropriate weights to each input feature. In this network, fast normalized feature fusion will be used as a weighting method as shown in the following equations:

(1) $$O=\sum_{i}\frac{w_{i}}{\in +\sum_{j}w_{j}}\cdot l_{i}$$

where $w_{i}$ is required to be non-negative ($w_{i}\geq 0)$, so a ReLU (Rectified Linear Unit) activation function is subsequently applied to ensure nonlinear processing. To alleviate the numerical instability problem, a minimum value ∊=0.0001 is specifically introduced. Ultimately, the BiFPN achieves an effective integration of features under the combined influence of bidirectional connectivity and a normalized fusion approach. Specifically, the fused features in layers 3 and 4 in BiFPN can be expressed as shown in the following equations, respectively.

(2) $$P_{n}^{td}=Block(\frac{w_{1}\cdot P_{n}^{in}+w_{2}\cdot Resize(P_{n+1}^{in})}{w_{1}+w_{2}+∊})$$

(3) $$P_{n}^{out}=Block\left(\frac{w_{1}^{\prime }\cdot P_{n}^{in}+w_{2}^{\prime }\cdot P_{n}^{td}+w_{3}^{\prime }\cdot Resize(P_{n-1}^{out})}{w_{1+}^{\prime }w_{2}^{\prime }+w_{3}^{\prime }+∊}\right)$$

In this context, for top-down paths, $P_{3}^{td}$ refers to the intermediate feature representation on layer 3, which is located on that particular layer of the path; correspondingly, on layer 3 of bottom-up paths, $P_{3}^{out}$ represents the output feature, which is the result of the path on that layer. Similarly, $P_{4}^{td}$ refers to the intermediate features on layer 4 of the top-down path, while $P_{4}^{out}$ represents the output features on layer 4 of the bottom-up path. The term “Resize” refers to the up-sampling or down-sampling operation performed. In addition, “Block” refers to the feature processing convolutional operations.

#### 3.2.3. Mixed Local Channel Attention

The growth of flowers in the context of the complex background of the flowering Chinese cabbage plant exhibits a high degree of irregularity and morphological diversity. This is evident not only in the differences in the size of flowers at different stages of growth but also in the size and morphology of flowers between plants at the same stage of maturity. Furthermore, the growth direction of flowers is haphazard and disorganized, and the perspective effect during imaging is influenced by the perceived target distance, resulting in significant alterations in the morphology, rotation angle and spatial position of objects within the field of view. However, the primitive network is constrained in its capacity to process spatial transformation information. This presents a significant challenge for accurately detecting their maturity. Many current channel attention mechanisms exclude spatial feature information, which limits the model’s performance in feature representation and target detection tasks. Moreover, the spatial attention module is often not used in practical applications because it tends to increase the computational complexity. For these issues, it may be more effective if the model fuses the channel and information of spatial and local features [35].

**Figure 10:**
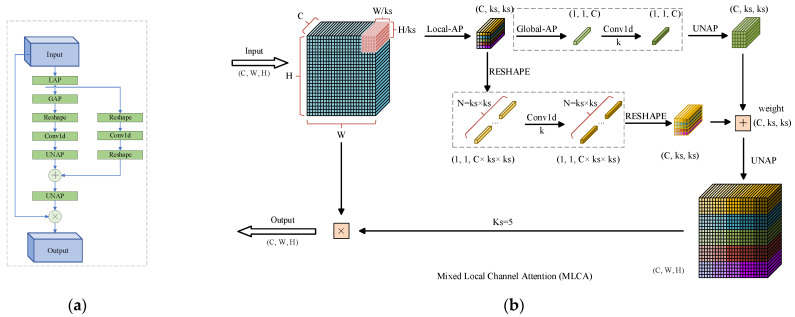
Mixed Local Channel Attention (MLCA): (**a**) Structural diagram; (**b**) Schematic diagram.

Accordingly, this study proposes the incorporation of the MLCA (Mixed Local Channel Attention) mechanism into the C2F module. The module depicted in Figure 10 illustrates the underlying principle of MLCA. The input feature vectors of MLCA undergo two pooling steps. Initially, the input is transformed into a vector of dimensions 1∗C∗ks∗ks, facilitating the extraction of local spatial information through the initial local pooling operation. Building upon the initial stage, the input is transformed into a vector through two distinct branches. which contains global information and local spatial information. Subsequently, one-dimensional convolution is applied, followed by inverse pooling to restore the original resolution of the individual vectors. Finally, the information from both branches is fused to facilitate hybrid attention. The size of the convolution kernel, denoted as “*k*”, is proportional to the channel dimension *C*. This implies that the local cross-channel interaction information is captured by considering only the relationship between each channel and its *k* neighboring channels. The selection of *k* is determined according to Equation (4) [36]:

(4) $$k=\Phi \left(C\right)={\left|\frac{\log_{2}\left(C\right)}{\gamma }+\frac{b}{\gamma }\right|}_{odd}$$

where *C* denotes the number of channels, *k* represents the size of the convolution kernel, *γ* and *b* are hyperparameters with a default value of 2, and the term “*odd*” specifies that k should be an odd number. If *k* is even, 1 is added to ensure it becomes odd.

MLCA is able to combine channel and spatial information, as well as local and global information simultaneously. This comprehensive information integration helps the model to better capture the features of small-sized targets, while the spatial attention mechanisms help the model to distinguish subtle differences between different targets and focus on critical regions in the image, even if these regions are occluded by other targets. And by suppressing irrelevant background information, the model can locate the occluded targets more accurately.

#### 3.2.4. Wise-Inner-IoU

During the experiments, we observed that although high-quality training samples can enhance the network fitting ability, it is also easy for the model to ignore the potential effects of low-quality samples, especially the uneven distribution of sample quality due to the large morphological changes and labeling subjectivity during the growth of flowering Chinese cabbage. Blind bounding box regression (BBR) enhancement of low-quality samples may impair localization performance. Existing BBR methods lack the flexibility to adapt to different detectors and tasks [37]. More importantly, existing bounding box regression methods often lack sufficient flexibility to adapt themselves to different detectors and tasks and are not very generalizable [38].

In order to overcome the limitations of the existing bounding box regression (BBR) loss function, this paper proposes a method that integrates two loss functions, Wise-IoU and Inner-IoU, and their related strategies, and expects to construct a more comprehensive and efficient one by combining the dynamic non-monotonic FM of Wise-IoU and the meticulous evaluation of the anchor frame quality of the Inner-IoU loss function to further enhance the performance of the target detection model.

In this paper, Wise-IoU version 3 (WIoU-v3), which introduces a dynamic non-monotonic focusing mechanism (FM), is used, and the principle is as follows: the degree of the outlier of the anchor box is characterized by the ratio of $L_{IoU}^{*}$ to $\bar{L_{IoU}}$ as shown in the following equation:

(5) $$\beta =\frac{L_{IoU}^{*}}{\bar{L_{IoU}}}\in [0,+\infty )$$

where $\bar{L_{IoU}}$ is the exponential running average with momentum. The anchor frame is of high quality and has a small outlier. To prioritize anchor frames of average quality for the bounding box regression (BBR), we assign a small gradient gain to such frames. Furthermore, anchor frames with significant outliers are assigned a reduced gradient gain, which mitigates the generation of large, detrimental gradients from low-quality examples effectively. Non-monotonic focusing factors using β are constructed and applied to Wise-IoU version 1 (WIoU v1) as shown in the following equation:

(6) $$L_{WIoUv3}=rL_{WIoUv1},\;r=\frac{\beta }{\delta \alpha^{\beta -\delta }}$$

where δ makes r = 1 when β=δ. The anchor frames receive the maximum gain of gradient when their outliers satisfy β=C (*C* is a constant value). Since $\bar{L_{IoU}}$ and the quality classification criteria of anchor frames are dynamic, so WIoU v3 is designed to facilitate the allocation of gradient gains in a manner that best adapts to the current situation at every moment.

**Figure 11:**
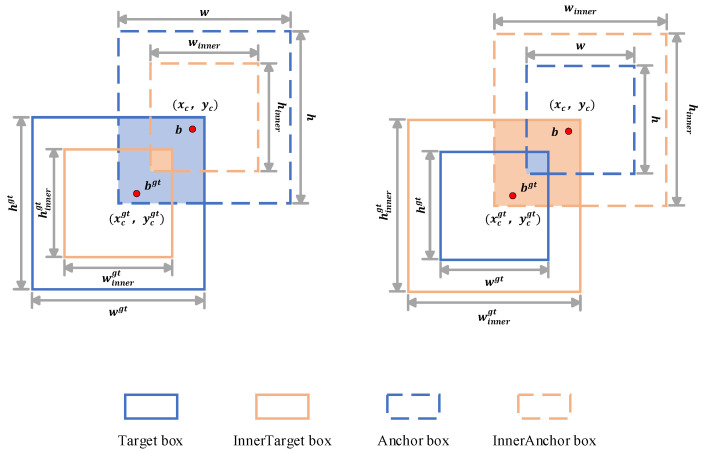
Inner-IoU.

In the Inner-IoU method, we introduce a core scale factor ratio (“ratio”), which provides flexibility in the size adjustment of the auxiliary bounding box. The issue of restricted generalization capacity, a common limitation of existing methods, is addressed by employing auxiliary bounding boxes of varying scales for diverse datasets and detector specifications. Specifically, given the anchor denoted as B and ground truth (GT) box denoted as $B^{gt}$, as illustrated in Figure 11, the center (red dot) of the GT box is identified as ($x_{c}^{gt},y_{c}^{gt}$), whereas the center (red dot) of the anchor is labeled as ($x_{c},y_{c}$). The width and height of the GT box are, respectively, represented as $w^{gt}$ and $h^{gt}$, while w and h denotes the width and height of the anchor. To make sure that the size and shape of targets in different datasets match more efficiently, a scale factor variable called “ratio” is defined, whose value is usually set within a reasonable range value [0.5, 1.5]. By adjusting the ratio factor, the size of the auxiliary bounding box can be controlled in a flexible manner, thereby optimizing the performance of the detector on targets of different sizes and shapes. This results in an improved overall detection effect.

Consequently, the application of the Inner-IoU loss to the Wise-IoU bounding box regression loss function yields the following equation:

(7) $$L_{Inner-WIoUv3}=L_{WIoUv3}+IoU-{IoU}^{inner}$$

The auxiliary bounding box computational loss at different scales facilitates the convergence process, while the dynamic allocation strategy of the Wise-IoU gradient gain effectively mitigates the detrimental effect of low-quality samples, thus improving the detection precision and effectiveness of the network. Introduction of an auxiliary bounding box, a scaled-down version of the original, is vital for optimizing loss functions in complex scenes with small, overlapping and occluded targets. It focuses on overlapping regions, enhancing accuracy in highly overlapping scenarios. Fine-tuning its scale enables Inner-IoU to concentrate on small targets’ cores, mitigating background noise and boosting small target detection. At the same time, combined with the dynamic focusing mechanism of Wise-IoU, the loss function can intelligently adjust its focus so that the model focuses more on targets or regions that are difficult to distinguish during the training process, such as highly similar or heavily occluded targets.

### 3.3. Tracking Based on ByteTrack

The inconsistency in the maturity period of flowering Chinese cabbage crops due to differences in growth cycles requires improved real-time detection capability of the model to achieve mobile scanning detection and synchronized harvesting to avoid economic losses. It is difficult to use methods that rely only on target detection and image classification algorithms to accurately reflect the actual ripening state and location of flowering Chinese cabbage in real time when facing the dynamically changing field environment, thus limiting the effectiveness of automated mechanical harvesting. In order to accurately detect the category and location of flowering Chinese cabbage during the mobile detection process, this study introduced the ByteTrack [39] target tracking algorithm to solve the above problems, aiming to achieve continuous and stable tracking of the flowering Chinese cabbage plants in complex dynamic scenes and to ensure that the ripening status of each flowering Chinese cabbage plant is continuously monitored during the mobile scanning process. Meanwhile, to enhance the detection accuracy and spatial localization capability, this study adopted the Intel RealSense D435i depth camera (Intel D435i depth camera is from Intel Corporation, Santa Clara, CA, USA) as the data acquisition device, which is capable of real-time capturing and extracting the 3D spatial information of the flowering Chinese cabbage plants, including the depth, distance and other key parameters.

**Figure 12:**
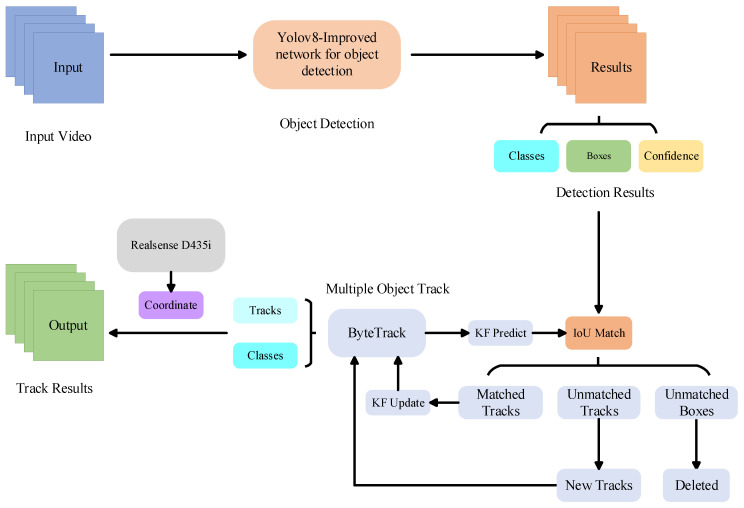
The structure of object detection and tracking based on video.

In the context of monitoring Chinese flowering cabbages, ByteTrack, a detection-based tracking (DBT) algorithm, is employed to generate tracks corresponding to individual cabbages. These tracks are subsequently matched frame-by-frame with the detection results produced by the YOLOv8-Improved model, enabling effective object tracking. The overall workflow of the model is depicted in Figure 12. In this context, the term “tracks” refers to the data representing the trajectories of objects. The ByteTrack algorithm categorizes the detection boxes generated by the YOLOv8-Improved model into two distinct categories: high-score boxes and low-score boxes. These categories are determined based on the confidence scores associated with each box. Additionally, ByteTrack divides the tracks into two types: long-term tracks and short-term tracks. Subsequently, the long-term tracks are matched with the high-score boxes, which have been identified through the application of the Kalman filter [40]. A successful match results in an immediate update of the position, whereas a failed match leads to the subsequent matching with low-score boxes.

Prior to the advent of ByteTrack, conventional detection-based tracking (DBT) algorithms placed exclusive reliance on high-scoring detection boxes, effectively discarding low-scoring ones. This approach frequently resulted in the loss of genuine targets and the fragmentation of trajectories. ByteTrack transformed this methodology by incorporating all detection boxes, including those with low scores, into the tracking process. This method not only enhanced the overall performance of the tracker but also addressed issues such as transient target disappearance which can be caused by occlusion, motion blur and other challenges. Furthermore, by incorporating low-scoring boxes, ByteTrack mitigated the issues of trajectory fragmentation and frequent ID switches that often occur due to low target confidence scores. Finally, ByteTrack achieved this enhancement without compromising on tracking speed, demonstrating its exceptional efficiency and reliability in real-world applications.

### 3.4. Evaluation Metrics

Performance metrics are utilized to comprehensively evaluate the model’s performance: precision (*P*), recall (*R*), average precision (*AP*) and comprehensive evaluation index (*F*1). These are defined in accordance with the equations presented in (8)–(11):

(8) $$Precision=\frac{TP}{\;TP+FP}\times 100\%$$

(9) $$Recall=\frac{TP}{\;TP+FN}\times 100\%$$

(10) $$AP=\int_{0}^{1}p\left(r\right)dr$$

(11) $$F1=2\times \frac{Precision\times Recall}{Precision+Recall}$$

The term “true positives” (*TP*) is used to indicate the number of target object samples that are correctly predicted by the model. In contrast, the term “false negatives” (*FN*) is used to indicate the number of target object samples that are incorrectly identified by the model. In contrast, false positives (*FP*) represent the number of instances where the model erroneously identifies non-target objects as target objects. Precision–recall (*PR*) curves were constructed by calculating a series of precision and recall values for varying confidence thresholds. Subsequently, the precision values for each recall were integrated along the precision–recall curve, resulting in the calculation of the average precision (*AP*) [41].

The computational complexity and performance of the model are measured using *GFlops* (giga floating point operations per second):

(12) $$GFlops=\frac{Nflops/10^{9}}{T}$$

(13) $$FPS=\frac{1}{T_{100}}$$

where *Nflops* denotes the overall count of floating-point operations executed by the model, which contains addition, multiplication and division. Analogously, the time required to execute the model and the total time required to make inferences of 100 images are denoted by *T* and *T*_100_, respectively, both quantified in seconds.

## 4. Discussion

In this study, we carefully constructed a diversified dataset to comprehensively cover the types of data characteristics under different environmental conditions to ensure the generalization ability of the model. Based on the biological characteristics and growth cycle of the target plants, we innovatively divided them into four growth stages, which laid a solid foundation for the subsequent model training and evaluation. Through a series of rigorous comparative experiments, the results showed that the YOLOv8-improved model achieves a significant improvement in detection accuracy and demonstrates excellent detection capability, which fully validates the robustness and effectiveness of the model in complex agricultural environments. Furthermore, we integrated the YOLOv8-Improved model with the ByteTrack tracking algorithm, which strengthens the real-time detection ability of the model in video streams and achieves accurate localization and continuous tracking of the target object by relying on the RGB-D camera, providing strong technical support for the intelligent operation of the harvesting robot. The experimental data show that the integrated system can meet the strict real-time requirements of harvesting operations while ensuring high detection accuracy.

**Figure 13:**
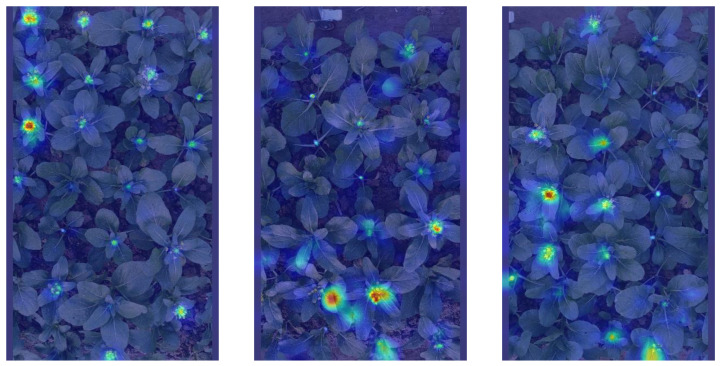
Heat map.

While this study has achieved notable advancements in detecting the maturity of flowering Chinese cabbage, it is crucial to acknowledge that there is potential for enhancing the system’s performance, and the present research has the following limitations: (1) Improving the interpretability of the model. Given the interpretability challenges associated with convolutional neural networks, it is recommended that visualization tools such as heatmaps be employed to parse the model’s decision-making basis during the detection process. This approach allows for the assessment of the impact of different locations on the detection efficacy of flowering Chinese cabbage. As illustrated in Figure 13, the degree of red hue intensity in the heatmap correlates with its influence on target detection. (2) The YOLOv8-Improved model performed well in detecting the vast majority of flowering Chinese cabbage, but still faced several challenges. Specifically, immature Chinese cabbage has limited detection accuracy due to its low pixel occupancy in the image, coupled with the high similarity between the shoots and the background and the fact that they are often occluded by the surrounding leaves. To solve this problem, future research will be devoted to refining the classification system by expanding the dataset containing samples from different growth periods to enhance the model’s ability to recognize subtle features. At the same time, the model structure is optimized to enhance its ability to extract and understand features in complex scenarios, aiming to reduce the loss of key semantic information so as to achieve more accurate detection of immature Chinese cabbage [42]. (3). This study preliminarily verifies the feasibility of real-time detection and preliminary estimation of the spatial location of flowering Chinese cabbage using RGB-D cameras, but the current 3D localization accuracy is not yet sufficient to support the demand for refined automated picking operations. Given that the core focus of this study is to improve the target detection accuracy, there are deficiencies in the in-depth exploration and experimental validation of the tracking algorithm. In the future, we will focus on combining the efficient target detection network with advanced tracking algorithms and optimize the algorithm design and parameter adjustments to achieve a seamless connection between detection and tracking tasks, so as to satisfy the needs of automatic harvesting systems for high-precision real-time tracking and detection of targets [43].

## 5. Conclusions

The real-time and accurate detection of the maturity of flowering Chinese cabbage flowers represents a crucial foundation for the development of an efficient and intelligent automated harvesting mechanical system for Chinese cabbage. Such a system would facilitate improvements in the efficiency of harvesting Chinese cabbage and the accuracy of crop quality management. To address this challenge, an innovative solution, designated as YOLOv8-Improved, is proposed in this study. The model proposed in this paper enhances the feature representation of channel and spatial dimensions by integrating the MLCA mechanism in the YOLOv8 network, weighting different channels and local regions to improve the model’s ability to recognize small-sized targets, to reduce leakage and misdetection, and to enhance the model’s ability to distinguish between similar targets; using BiFPN neck network and adding P2 detection layer, and by combining high-level semantic information and low-level detail information, the network can more accurately localize and identify occluded or overlapped targets and improve the detection accuracy of small-sized targets; adding the Wise-Inner-IoU loss function, which efficiently focuses on the core region of small-sized targets, combined with the dynamic focusing mechanism to intelligently adjust its focus, so that the model focuses more on those hard-to-distinguish targets or regions during the training process. These improvements markedly enhance the model’s capacity to detect multi-target, multi-category and multi-size Chinese flowering cabbages, with precision and recall reaching 86.0% and 86.5%, respectively, and mAP_50_. The mAP_75_ reached 91.8% and 61.6%, respectively, with a more pronounced improvement in model parameters, which decreased by 25.34%. The model demonstrates remarkable detection capabilities across all stages of Chinese flowering cabbage growth, facilitating real-time monitoring of the crop’s condition and assessment of its maturity. This precise information enables the automated harvesting machinery to periodically select only the ripened cabbage, preventing economic loss due to over-ripeness and unharvested crops.

## Figures and Tables

**Table 1:** Comparison of the performance among various network models. (The best data for that column is shown in bold).

Model	Precision (%)	Recall (%)	F1	mAP_50_ (%)	GFlops (G)
Faster R-CNN	70.7	81.5	0.76	80.1	132.7
SSD	78.3	55.9	0.65	75.7	274.9
RetinaNet	72.1	**88.5**	0.80	87.3	128.3
YOLOv5	83.1	83.3	0.83	88.0	**4.1**
YOLOv8	84.9	83.3	0.84	88.9	8.1
YOLOv9	79.3	74.6	0.77	81.1	18.2
RT-DETR	66.6	66.9	0.67	68.9	100.6
YOLOv8-Improved	**86.5**	86.0	**0.86**	**91.8**	16.5

**Table 2:** Comparison results of different models for ablation experiments. (The best data for that column is shown in bold).

Tags	W	X	Y	Z	P (%)	R (%)	mAP_50_ (%)	mAP_75_ (%)	GFlops (G)	Params (M)
1					84.9	83.3	88.9	56.9	**8.1**	3.01
2	**√**				84.0	85.7	90.1	58.7	12.2	2.92
3	**√**	**√**			85.6	85.9	90.7	59.2	12.2	2.92
4	**√**		**√**		85.3	**86.8**	91.4	59.1	16.5	2.18
5	**√**			**√**	84.0	86.4	90.3	59.2	12.2	2.92
6	**√**	**√**	**√**	**√**	**86.5**	86.0	**91.8**	**61.6**	16.5	**2.18**

**Table 3:** Comparison experiment of attention mechanism parameters. (The best data for that column is shown in bold).

Size	Weight	Precision (%)	Recall (%)	mAP_50_ (%)
5	0.3	85.2	85.5	90.1
5	0.5	**86.5**	86.0	**91.8**
5	0.7	84.3	87.3	90.7
5	1.0	84.7	87.3	91.0
3	0.5	83.3	**87.4**	90.3
7	0.5	84.8	84.8	90.5

**Table 4:** Data samples of different varieties of flowering Chinese cabbage.

States	Training	Validation	Test	Total
Growing	2868	781	434	4063
Ripe	1901	499	314	2714
Over-Ripe	1608	411	259	2278
Harvested	1991	579	325	2895
Model samples	8368	2270	1332	11,970

## Data Availability

Data are contained within the article.

## Abbreviations

The following abbreviations are used in this manuscript:

RGB-D	Red, Green, Blue, and Depth
MLCA	Mixed Local Channel Attention
BiFPN	Bidirectional Feature Pyramid Network
ReLU	Rectified Linear Unit
IoU	Intersection over Union
DFL	Distributional Feature Loss
FPS	Frames Per Second
Params	Parameters
GFlops	Giga Floating-point Operations Per Second
TP	True Positive
FP	False Positive
FN	False Negative
P	Precision
R	Recall

## Appendix A

The appendix to this section contains the confusion matrix of various models in the ablation experiment, which is presented here to ensure the fluency of the article.
